# Duodenal gangliocytic paraganglioma showing lymph node metastasis: A rare case report

**DOI:** 10.1186/1746-1596-5-27

**Published:** 2010-05-06

**Authors:** Yoichiro Okubo, Tomoyuki Yokose, Masaru Tuchiya, Aki Mituda, Megumi Wakayama, Chikako Hasegawa, Daisuke Sasai, Tetsuo Nemoto, Kazutoshi Shibuya

**Affiliations:** 1 Department of Surgical Pathology, Toho University School of Medicine, 6-11-1 Omori-Nishi, Ota-Ku, Tokyo, 143-8541, Japan; 2 Department of Pathology, Kanagawa Cancer Center, 1-1-2, Nakao, Asahi-Ku, Yokohama-city, Kanagawa, 245-0815, Japan; 3 Division of General and Gastroenterological Surgery, Department of Surgery (Omori), Toho University School of Medicine, 6-11-1 Omori-Nishi, Ota-Ku, Tokyo, 143-8541, Japan; 4 Department of Pathology, Tokyo Metropolitan Cancer and Infectious Diseases Center, Komagome Hospital, 18-22, Honkomagome 3 chome, Bunkyo-Ku, Tokyo, 113-8677, Japan

## Abstract

We describe a case of duodenal gangliocytic paraganglioma showing lymph node metastasis. A 61-year-old Japanese man underwent pylorus preserving pancreaticoduodenectomy to remove a tumor at the papilla of Vater. The section of the tumor extending from the mucosa to submucosa of the duodenum was sharply demarcated, solid, and white-yellowish. Neither necrosis nor hemorrhage was present. Histological examination confirmed the immunohistochemical identification of three components comprising epithelioid cells, spindle-shaped cells, and ganglion-like cells. Epithelioid cells showed positive reactivity for synaptophysin, somatostatin, and CD56. In contrast, spindle-shaped cells showed positive reactivity for S-100 protein, but not for synaptophysin, somatostatin or CD56. Furthermore, we found lymph node metastasis despite lack of bcl-2 and p53 expression. In addition to the rarity of the tumor, we are describing here the present case suggests the malignant potency of the tumor despite lack of acceptable prognostic indicators for neuroendocrine tumor.

## Background

Gangliocytic paraganglioma is a rare neuroendocrine tumor that usually occurs at the second portion of the duodenum, and its ultimate diagnosis requires a histopathological identification of three identical components comprising epithelioid cells, spindle-shaped cells, and ganglion-like cells [1]. This tumor has been regarded as benign in general, but a few cases with lymph node metastasis have been reported which required extensive surgical removal [Table 1]. In addition to the rarity of the tumor, the present case suggests the malignant potency of the tumor despite lack of bcl-2 and p53 expression, which has been known as a marker for malignancy of neuroendocrine tumors [2-5].

**Table 1:** Gangliocytic paraganglioma showing lymph node metastasis

Reference	Year	Age (years)	Sex	Chief Clinical presentation	Site	Size (mm)	Operation	Follow up (months)
Inai et al. [16]	1989	17	Male	Hematoemesis	Papilla of Vater	20	PD	NED 32
Hashimoto et al. [17]	1992	47	Male	Incidental findings	Papilla of Vater	65	PD	NED 14
Takabayashi et al. [18]	1993	63	Female	Abdominal pain	Papilla of Vater	32	PPPD	NED 24
Tomic et al. [19]	1996	74	Female	Abdominal pain, vomiting, weight loss	Pancreas	40	PD	NED 19
Sundararajan et al. [1]	2003	67	Female	Incidental findings	Second part of duodenum	50	PD	NED 9
Bucher et al. [20]	2004	31	Female	Anemia, subclinical jaundice	Papilla of Vater	30	PPPD	NED 44
Wong et al. [10]	2005	49	Female	Melena	Duodenum	14	PPPD	NED 12
Witkiewicz et al. [21]	2007	38	Female	Abdominal pain	Papilla of Vater	15	PPPD	NR
Mann et al. [22]	2009	17	Female	Abdominal pain, vomiting, weight loss	Duodenum	NR	PPPD	NR
Present case	2010	61	Male	Epigastralgia, tarry stool	Papilla of Vater	30	PPPD	NED 6

NR: not reported, PD: pancreatoduodenectomy, PPPD: pylorus-preserving pancreaticoduodenectomy

NED: no evidence of disease

There are ten cases of gangliocytic paraganglioma showing lymph node metastasis including the present case.

## Case presentation

A 61-year-old Japanese man presented with epigastralgia and tarry stool a month before admission. He had history of neither habitual smoking nor irradiation. A gastrointestinal endoscopy revealed a tumor with central ulceration at the papilla of Vater. He was referred to our hospital after an endoscopic procedure for the bleeding. Subsequent examinations in our hospital included upper gastrointestinal endoscopy, magnetic resonance cholangiopancreatography, endoscopic retrograde cholangiopancreatography, and endoscopic ultrasonography, which led to the detection of a tumor at the papilla of Vater that suggested regional lymph node metastasis. A biopsy before the operation suggested a duodenal carcinoid following histological findings and the results of immunohistochemical examination. The tumor cells showed positive reactivity for synaptophysin, somatostatin, and CD56. The patient underwent pylorus preserving pancreaticoduodenectomy with lymph nodes dissection.

## Pathological findings

**Figure 1:**
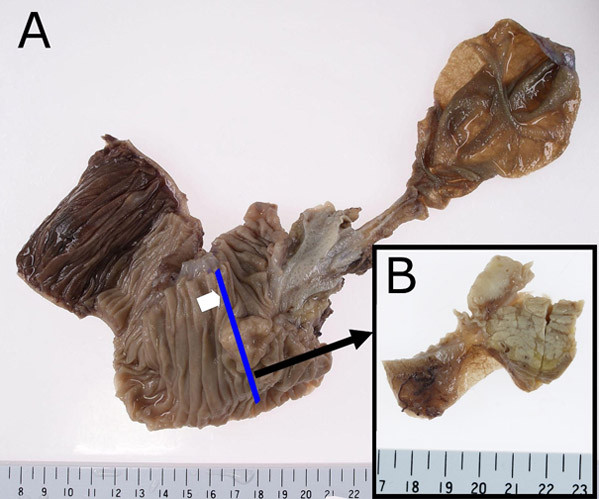
**The surgical specimen**. (A) A solid tumor measuring 25 × 30 × 25 mm in size was found at the papilla of Vater which was lobulated and covered by attenuated mucosa with ulcer formation at the center of elevation. (B) The section of the tumor extending from the mucosa to submucosa of the duodenum was sharply demarcated, solid, and white-yellowish.

**Figure 2:**
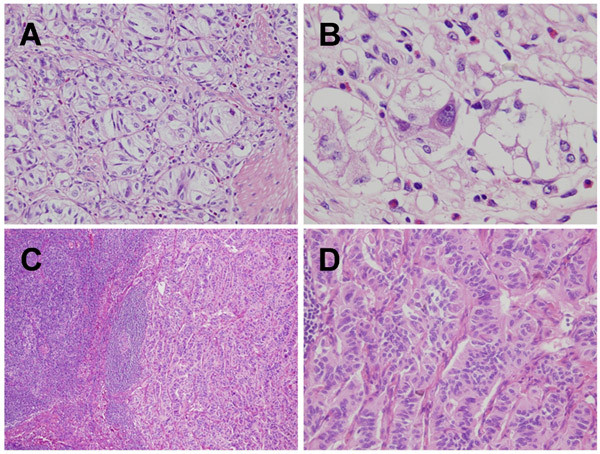
**Photomicrographs showing different types of tumor cells**. (A) Tumor cells of epithelioid cell type are usually nested which are encompassed by those of spindle-shaped cell type with alignment of a single cell layer (HE double stain, × 400). (B) Ganglion-like cells; third type of the tumor cell had a large and oval nucleus with conspicuous nucleolus, as well as a polyhedral amphophilic cytoplasm (HE double stain, × 1000). (C and D) The metastasizing component of the tumor at the lymph node comprised the epithelioid cell type alone (HE double stain, × 100, × 400, respectively).

The surgical specimen, an en-bloc comprising duodenum, bile duct, gallbladder, and head of pancreas, was fixed with 10% buffered formalin. A solid tumor 25 × 30 × 25 mm in size was found at the papilla of Vater whose surface was lobulated and covered by attenuated mucosa showing ulcer formation at the center of elevation (Fig. 1A). The section of the tumor extending from the mucosa to submucosa of the duodenum was sharply demarcated, solid and white-yellowish (Fig. 1B). Neither necrosis nor hemorrhage was present. Sections of paraffin-embedded tissue were prepared and stained with hematoxylin and eosin (HE) double stain for light microscopic observation. Histological examination showed that a large body of the tumor was present in the submucosa and invaded a part of the muscularis propria, but the bile duct and pancreas were not involved. Histological observation also revealed that the tumor consisted of three identical cellular components: epithelioid cells, spindle-shaped cells, and ganglion-like cells. Tumor cells of an epithelioid cell type usually nested and had a round to oval-shaped nucleus with an inconspicuous nucleolus, as well as a clear and eosinophilic cytoplasm (Fig. 2A). Tumor cells of a spindle-shaped cell type encompassed the nests of epithelioid cells with alignment of a single cell layer and had an elongated and plump nucleus, including an attenuated eosinophilic cytoplasm (Fig. 2A). Tumor cells of a ganglion-like cell type were rarely seen and had a round nucleus with conspicuous nucleolus, as well as a polyhedral amphophilic cytoplasm (Fig. 2B). In addition to the absence of mitosis among these cells, neither necrosis nor hemorrhage was found. However, tumor cells of an epithelioid cell type showed regional lymph node metastasis (Fig. 2C and 2D).

## Immunohistochemical findings

**Figure 3:**
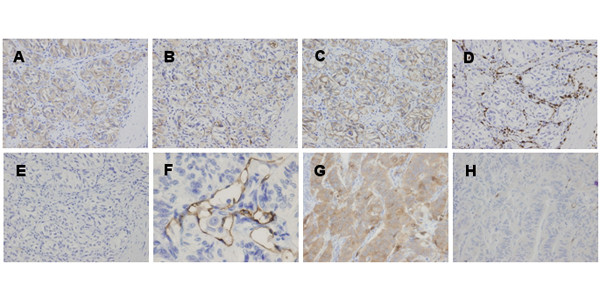
**Photomicrographs showing representative immunohistochemistry**. (A, B, and C) Epithelioid cells showed positive reactivity for synaptophysin, somatostatin, and CD56 (× 400). (D) Spindle-shaped cells showed positive reactivity for S-100 protein (× 400). (E) Tumor cells of both types showed negative reactivity for chromogranin A (× 400). (F) Tumor cells invade into the lymphatic lumen (immunohistochemistry with podoplanin (D2-40) antibody, × 400). (G) Epithelioid cells metastasizing at the lymph node showed positive reactivity for synaptophysin (× 400). (H) No spindle-shaped cells were found in the metastatic focus as confirmed by the lack of S-100 protein-positive cells (× 400).

**Figure 4:**
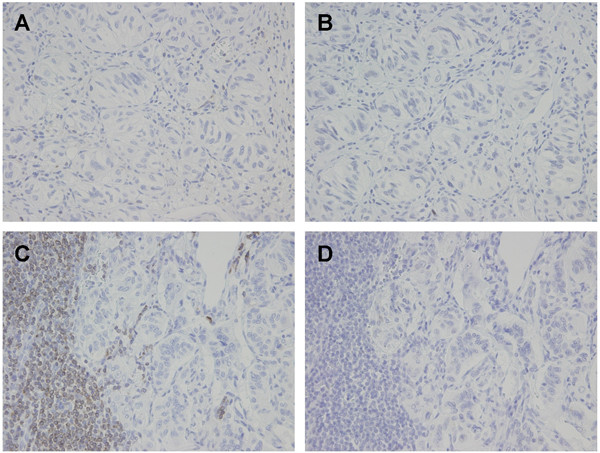
**Photomicrographs showing immunohistochemistry for bcl-2 and p53**. (A and B) Tumor cells in primary focus showed negative reactivity for both bcl-2 and p53 (× 400). (C and D) Tumor cells in metastatic focus showed negative reactivity for both bcl-2 and p53 (× 400).

Several kinds of antibodies were used to evaluate and identify tumor cells immunohistochemically. Tumor cells of both epithelioid and ganglion-like cell types showed positive reactivity for synaptophysin, somatostatin, and CD56 (Fig. 3A, B, and 3C). In contrast, the spindle-shaped cell type showed positive reactivity for S-100 protein (Fig. 3D), but not for synaptophysin, somatostatin or CD56. Neither cell type was positive for chromogranin A (Fig. 3E), cytokeratin AE1/AE3 or epithelial membrane antigen (EMA). MIB-1 (Ki-67) labeling index estimated less than 1% in both primary and metastatic foci. Tumor cells invade into the lymphatic lumen which can be recognized by an enclose of podoplanin (D2-40)-positive endothelial cell (Fig. 3F). Tumor cells of the epithelioid cell type metastasizing at the lymph node showed positive reactivity for synaptophysin (Fig. 3G). No tumor cells of the spindle-shaped cell type were found in the metastatic focus as confirmed by the lack of S-100 protein-positive cells (Fig. 3H). Furthermore, tumor cells of the epithelioid cell type in both primary and metastatic foci showed negative reactivity for both bcl-2 and p53 in the present case (Fig. 4A, B, C, and 4D).

## Discussion

Gangliocytic paraganglioma has been known as a rare and benign neuroendocrine tumor. This condition occurs at the gastrointestinal tract, especially at the second portion of the duodenum [1]. Major clinical symptoms of the disease include abdominal pain, nausea, vomiting, and gastrointestinal bleeding. Biliary obstruction has rarely been reported as a devastating complication [6,7]. Histopathological diagnosis of the disease should require a confirmation of three identical components comprising epithelioid cells, spindle-shaped cells, and ganglion-like cells [1,6-8]. Carcinoid tumor, paraganglioma, ganglioneuroma or spindle cell tumors (including nerve sheath tumors, smooth muscle tumors and gastrointestinal stromal tumors) should be referred as diseases for differential diagnosis in cases of histopathological investigation [6]. To identify the three cellular components, in addition to the detailed microscopic examination of hematoxylin and eosin double stained sections, phenotypical expression analysis by immunohistochemical examination has been regarded as an important diagnostic clue [8,9]. Although gangliocytic paraganglioma has been regarded as a benign tumor, we found nine cases showing lymph node metastasis among the literature [Table 1]. The table surmises major clinical data of ten cases of gangliocytic paraganglioma with lymph node metastasis including the present case were analyzed. Patient age ranged from 17 to 74 (mean: 46.4). Male-to-female ratio was 3:7. Tumor sizes ranged from 14 to 65 mm (mean: 32.9). Nine cases occurred at duodenum, but one occurred at pancreas. Although present patient was older, its location and size follows characteristic manner of the disease. One case had irradiation as an adjuvant therapy [10], no recurrence or distant metastasis, however, was described in all patients who we had extracted from the previously reported cases. Therefore, we decided to avoid any adjuvant therapy from the present case. The results of immunohistochemical examinations are summarized in Table 2 with those of previously reported cases of gangliocytic paraganglioma with lymph node metastasis. In the present case, three identical components were clearly identified by phenotypical expression analysis, which correspond to those previously reported with the exception of positive reactivity for chromogranin A. In addition, the present case also showed lymphatic involvement of tumor cells of an epithelioid cell type at the primary focus and regional lymph node metastasis. Furthermore, there was a study suggesting approximately 5% of gangliocytic paraganglioma demonstrate malignant behavior [10], an importance of imaging examinations before an operation should be emphasize to determine the most suitable method of surgical intervention [11]. On the other hand, several studies conducting to neuroendocrine tumor have reported that bcl-2 and/or p53 expression might be correlated with malignant behavior of them [2-5], whereas no studies were found of which investigation was limited in gangliocytic paraganglioma.

**Table 2:** Summary of phenotypical expression analysis by immunohistochemical examination

	Literature summary*			Present case		
	**Epithelioid cell**	**Spindle-Shaped cell**	**Ganglion-like cell**	**Epithelioid cell**	**Spindle-Shaped cell**	**Ganglion-like cell**
CD56	NR	NR	NR	+	-	+
Chromogranin A	4/4	0/2	1/2	-	-	-
Somatostatin	3/3	0/1	2/2	+	-	+
Synaptophysin	4/4	1/3	3/3	+	-	+
S-100	1/2	6/6	0/2	-	+	-
CK	1/1	NR	NR	-	-	-
NF	1/2	2/3	1/2	ND	ND	ND
NSE	3/3	3/3	2/2	ND	ND	ND
PP	1/2	0/1	2/2	ND	ND	ND
bcl-2	NR	NR	NR	-	-	-
p53	NR	NR	NR	-	-	-

CK: Cytokeratin, NF: Neurofilament, NSE: Neuron-specific enolase, PP: Pancreatic polypeptide

NR: Not reported, ND: Not done in the present case.

*: Based on referenced [1,16-19], and [21].

In the present case, three identical components have been clearly identified by phenotypical expression analysis, which corresponds to those previously reported with the exception for chromogranin A. Furthermore, we found lymph node metastasis despite lack of bcl-2 and p53 expression.

Therefore, we unfortunately have to conclude an immunohistochemical evaluation using bcl-2 or p53 in gangliocytic paraganglioma has a limited prognostic value mostly due to its rarity. In fact, some studies indicated that bcl-2 and/or p53 expression was not correlated with malignant behavior in gastrointestinal neuroendocrine tumors [12,13]. In addition, MIB-1 (Ki-67) has been known as a prognostic indicator in neuroendocrine tumor [14]. However, none of previously reported cases of gangliocytic paraganglioma with lymph node metastasis described the MIB-1 (Ki-67) labeling index and that of the present case estimated less than 1% in both primary and metastatic foci. These finding suggests that immunohistochemical evaluation using MIB-1 (Ki-67) has also a limited prognostic value in gangliocytic paraganglioma.

In the meantime, Hagemeyer et al. reported the case of pulmonary paraganglioma with p53 expression after Tschernobyl radiation [15]. They suggested that the irradiation had little influence on development of pulmonary paraganglioma, but smoking or irradiation could be associated with p53 expression. A part of their notion may be supported because our patient showed no p53 expression who had history of neither smoking nor irradiation. However, it should be unripe that smoking or irradiation has a relationship to p53 expression of tumor cell in gangliocytic paraganglioma. We wish to emphasize the importance of imaging examinations to monitor for recurrence or metastasis after the operation, because appropriate prognostic indicators have not been accepted using immunohistochemical evaluation.

The operation of our patient resulted in success, and he still maintains a high quality of life six months after that. Neither chemotherapy nor radiation has started because consensus on the adjuvant therapy for this tumor has not been reached at present.

## Conclusions

We describe a case of duodenal gangliocytic paraganglioma showing lymph node metastasis, of which histological and immunohistochemical examinations revealed the tumor comprised three identical cellular components; epithelioid cells, spindle-shaped cells, and ganglion-like cells. Furthermore, the case showed node metastasis despite lack of bcl-2 and p53 expression.

In addition to the rarity of the tumor, we wish to emphasize that the malignant potency of the tumor despite lack of acceptable prognostic indicators for neuroendocrine tumor.

## Abbreviations

HE: hematoxylin and eosin; EMA: epithelial membrane antigen.

## Competing interests

Dr. Shibuya reports receiving research grants from Pfizer Inc., Janssen Pharmaceutical K.K., and Dainippon Sumitomo Pharma Co. All authors declare that they have no competing interests.

## Authors' contributions

YO conceptualized the case report, integrated the data, and wrote the manuscript as a major contributor; TY carried out the histopathology evaluation and revised histopathological description; MT performed operation and contributed to management of the patient; AM, MW, CH, DS, and TN carried out the histopathologic evaluation; KS gave final approval to the manuscript as a corresponding author. All authors contributed to conceptualizing and writing this case report. Furthermore, all authors read and approved the final manuscript.

## Consent

Written informed consent was obtained from the patient for publication of this case report and any accompanying images. A copy of the written consent is available for review by the Editor-in-Chief of this journal.
